# Prognosis of recurrent bacterial vaginosis based on longitudinal changes in abundance of *Lactobacillus* and specific species of *Gardnerella*

**DOI:** 10.1371/journal.pone.0256445

**Published:** 2021-08-23

**Authors:** Essence Turner, Jack D. Sobel, Robert A. Akins

**Affiliations:** 1 Department of Biochemistry, Microbiology, & Immunology, Wayne State University School of Medicine, Detroit, Michigan, United States of America; 2 Division of Infectious Diseases, Wayne State University School of Medicine, Detroit, Michigan, United States of America; University of Illinois Urbana-Champaign, UNITED STATES

## Abstract

Refractory responses to standard-of-care oral metronidazole among recurrent bacterial vaginosis (BV) patients is not rare, and recurrence within a year is common. A better understanding of the bacterial determinants of these outcomes is essential. In this study we ask whether changes in specific species of *Gardnerella* are associated with poor short or long term clinical outcomes, and if and how resurgence of *Lactobacillus* species affects these outcomes. We quantify *Lactobacillus* isolates as a proportion of total vaginal bacteria using the LbRC5 qPCR assay, and 5 prevalent species of *Gardnerella* using primers that target species-specific polymorphisms within the *cpn60* gene. The study includes 43 BV patients: 18 refractory, 16 recurrent, and 11 remission patients, sampled daily for up to two weeks post-treatment; clinical outcomes were tracked for up to 9 months. Persistently high titers of *Gardnerella Gsp07* were associated with refractory responses, and persistently low abundance of *Gardnerella Gsp07 and G*. *swidsinskii* / *G*. *leopoldii* were associated with remission. *Lactobacillus* species abundance rose in 4–14 days after initiation of treatment in most but not all recurrent and remission patients, although increases were more sustained among remission patients. The findings suggest that *Gardnerella Gsp07 and G*. *swidsinskii* / *G*. *leopoldii* are markers of poor clinical outcome or may directly or indirectly suppress recovery of *Lactobacillus* species, thereby interfering with clinical recovery. Therapies that target these strains may improve patient outcome.

## Introduction

The pathogenesis of BV is complex and still not fully understood. It is a polymicrobial phenomenon, and there may be many pathways to convert healthy microbiota to asymptomatic or symptomatic dysbiosis. *Lactobacillus* species (*L*. *crispatus*, *L*. *gasseri*, *L*. *jensenii*) are the typical but not universal dominant species of eubiosis, and *L*. *iners* is present, often at high levels, in both healthy women and BV patients [1–8]. In vitro and animal model studies show that some strains of *Lactobacillus* species are antagonistic to at least some strains of BV-associated bacteria, including *Gardnerella species* [9–14].

The role of *Gardnerella* in bacterial vaginosis has been controversial because it is often present in the healthy vaginal microbiome, typically but not always at lower titer, and because its higher titer in BV patients, while common, is not universal [1, 4, 5, 15–19]. It is generally hypothesized that these distributions are possible because of differences in virulence between clades, now species, of *Gardnerella* [20–23]. *Gardnerella* forms biofilm on the vaginal epithelium and may be the major instigator for the congregation of a complex population of BV-associated bacterial species which contribute to the symptoms of the disorder [24–27].

Molecular studies in the past 8 years have shown that *Gardnerella* isolates are a highly diverse collection of 4–13 groups, now recognized as distinct species. Isolates share only 200 core genes of 7402 genes in the pan-genome; the average isolate has a genome of 1374 genes, among 103 strains in the NCBI whole genome database [28]. Four clades of *Gardnerella* have been defined by sequencing polymorphisms within the *cpn60* gene [19, 29–32], and by qPCR detection of clade-specific genes (clades 1–4 or C1-C4) [33]. These clades had been delineated by a genome-based comparison which suggested these were genovars or distinct species [34]. More recently, two whole genome sequencing studies on an expanded inventory of genome sequences identified 9 genomospecies [28] or 13 *Gardnerella* species [35]. These genomic studies indicate that the subgroups each constitute a separate species of *Gardnerella*. A larger genome-based analysis of all bacterial taxonomy also supports that these subgroups of *Gardnerella* constitute separate species but proposes yet another nomenclature [36]. To avoid confusion, we will use the nomenclature of Vaneechoutte et al. [35], which we abbreviate as Gsp01-Gsp13, and summarize the relationships of these nomenclatures in Table 1. We place the 108 NCBI reference genomes into their various groups in S1 Fig and S1 Table.

**Table 1:** Classification systems of *Gardnerella* isolates.

Group	Clade	*Gardnerella* genomospecies	*Gardnerella* species
C	C1	*GS01*	*G*. *vaginalis (Gsp01)* & *Gsp02*
B	C2	*GS02*	*G*. *piotii* & *Gsp03*
D	C3	*GS05*	*Gsp08*,*Gsp09*, & *Gsp10*
		*GS04*	*Gsp07*
A	C4	*GS03*	*G*. *swidsinskii* & *G*. *leopoldii*
B		*GS06*	*Gsp11*
		*GS07*	*Gsp12*
		*GS08*	*Gsp13*
		*GS09*	

Classifications across lines in the table encompass approximately the same isolates. Groups A-D were assigned based on the phylogenetic relationships of a subsequence of the *cpn60* gene [31]. Clades were assigned based on the qPCR detection of 4 genes, each specific for 4 major branches of *Gardnerella* isolates [37], designated as genovars by genome sequencing of 17 isolates [34]. Genomospecies (GS) were defined by integrating 4 methods of sequence comparisons of 103 genomic sequences [28]. *Gardnerella* species, labelled with names or numbers, were defined by analysis of 81 genome sequences by digital DNA-DNA hybridization, average nucleotide identity (ANI) and by MALDI-MS protein signatures [35]. Detailed placements of reference isolates are found in S1 Fig and S1 Table.

Studies attempting to associate clades with virulence by measuring titer or prevalence in vaginal samples from healthy women versus BV patients, or with progression to recurrence after treatment, have yielded inconsistent results. Individual studies that used primers targeting clade-specific genes were inconsistent in that they found different subsets of clades associated with BV: C1 and C3 [37], C1 and C2 [19, 38], C4 [39], C1,C2,and C4 [40], or C1,C2, and C3 [41]. No study linked observed associations with causality. All these studies found that an increased number of clades per sample was associated with BV. Our study differs from these in our *cpn60*-based qPCR approach, which unlike clade-specific qPCR [37], allows detection of *Gsp07* and is more inclusive of *G*. *piotii* & *Gsp03* isolates. Our enrollment strategy differs by emphasizing long term clinical follow-up to allow classification by outcome. Furthermore, this study quantifies *Lactobacillus* species using the *Lactobacillus* Relative Content (LbRC5) assay [42, 43]. This assay performs side-by-side qPCR assays on a sample with broad-spectrum bacterial 16S ribosomal RNA primers; one of the assays includes 3’ phosphorylated oligomers that specifically complement *Lactobacillus* sp. sequences to interfere with primer binding or extension. The assay score is a composite of the change in Cq values between the two assays, with a penalty for detecting non-*Lactobacillus* Tm values. In essence it is a measure of the extent of dominance by *Lactobacillus* species over other vaginal species. A key advantage of this assay is that the duplicate assays both rely on the same broad-spectrum primers, so that inhibitors and inevitable primer-template mismatches impact the two assays to the same extent, providing internal normalization.

In addition to clinical association studies, in vitro studies have attempted to categorize clades into low versus high virulence types, again with mixed results, which may indicate that phenotypic diversity exists not just between clades but also among strains within clades. Most of these phenotypic studies were done before molecular clade typing systems and will not be addressed here; they have been reviewed recently [44]. Clade 2 isolates were uniquely and pervasively (9 of 9) positive for sialidase activity [29]. Confusion about the broader distribution of sialidase stemmed from the PCR based detection of the sialidase A gene; recent studies show that the major sialidase genes are encoded instead by NanH2 and NanH3, and reside only in clade 2 isolates [45, 46]. The issue of whether differences in susceptibility to metronidazole is clade-specific has also met with mixed results. One study characterized isolates belonging to C3 or C4 as 100% resistant *in vitro*, compared to only 43% of C1 isolates and 7% of C2 isolates [47]. In contrast, a metatranscriptome analysis of responses of BV patients to metronidazole showed that subgroups A and D (C4 and C3) were enriched about 2-fold among patients who did not respond well to therapy, whereas C1 and C2 were depleted in non-responders, suggesting that drug resistance among C4 and C3 isolates is more prevalent and associated with clinical outcome [38].

This study addresses the pathobiology of recurrence among highly recurrent BV patients, and offers a prognostic marker for poor clinical outcome of oral metronidazole therapy at presentation. It distinguished the patterns of recovery of *Lactobacillus* species after treatment among refractory, recurrent, and remission patients, and shows that higher abundance of *Gsp07* and *G*. *swidsinskii/G*. *leopoldii* shortly after therapy are markers of, or possibly contributors to poor clinical outcome.

## Materials and methods

### Patient cohort

This study utilized a subset of patients and collection times from an investigation we previously described [39, 48]. This was a single cross-over prospective pilot study, performed at a Vaginitis Clinic at Wayne State University in Detroit, MI, and enrolled RBV patients from September 30, 2014 to December 1, 2017. Participants were recruited by regionally distributed flyers and digital advertisements. Major requirements for enrollment as recurrent BV patients were: a history of ≥ 3 episodes of symptomatic BV in the previous year, positive at enrollment for ≥3 Amsel criteria (vaginal pH ≥ 4.5, positive amine “Whiff” test, > 20% clue cells, and grayish-white adherent discharge) [49], symptomatic (odor, discharge, discomfort or itching), premenopausal, ≥18 years old, heterosexual, no mixed vaginal infections, willing to refrain from using any other vaginal products during the study period, willing to either use condoms for the duration of the study or to report unprotected sex, willing to abstain from coitus within 48 hours of any study visit, and willing to abstain from alcohol during therapy. Symptomatic patients with Amsel-confirmed BV were prescribed oral metronidazole 500 mg bid for 7 days (standard of care, SOC) and seen on average 16 days following initiating therapy and monthly for up to 9 months. Patients returning at the next (second) visit after SOC therapy with symptoms of BV and ≥3 Amsel criteria were classified as refractory patients. Those who were in clinical remission at this second visit, but later returned to clinic with symptoms and ≥3 Amsel criteria were classified as recurrent patients. Those who never again developed symptoms or ≥3 Amsel criteria and who stayed in the study for at least 3 months were classified as remission patients. Patients were followed for up to 9 months unless they acquired other exclusion criteria. In addition to monthly exams in the clinic, all patients also performed daily vaginal self-swabs following provided instructions, and recorded whether they engaged in sex, with or without a condom, and when they experienced menses. The protocol was approved by Wayne State University Institutional Review Board (IRB 040314M1F) and enrolled patients with written informed consent in compliance with the Declaration of Helsinki; the full protocol is posted online.

In this study we focused on the initial phase of treatment, the 7 days during which patients took oral metronidazole, and the approximately 7 days thereafter until their second clinic visit. Patients were classified as refractory, recurrent, or remission based on the longer term criteria described above, so that recurrent patients at the second visit were in clinical remission but recurred at a later date. This included 18 refractory patients, 16 recurrent patients, and 11 remission patients as characterized in Table 2. This cohort of participants is representative of highly recurrent, largely African American patients across the country.

**Table 2:** Characteristics of BV patients and outcome groups at enrollment.

	Refractory	Recurrent	Remission	P value
Patient Numbers	18	16	11	
African American	17	16	7	
Caucasian	1	0	4	
Days	16 (5.3)	50 (17.2)	209 (124)	<0.0001
Age	34 (7.8)	34 (6.2)	35 (6.9)	0.843
pH	5.7 (0.3)	5.6 (0.2)	5.2 (1.6)	0.726
Amsel	3.8 (0.4)	3.9 (0.3)	3.9 (0.3)	0.359
Nugent	8.7 (1.1)	8.3 (1.7)	8.6 (0.8)	0.698

Mean (**standard deviation**) of patients at enrollment. Days indicate days from enrollment to a first diagnosis of symptomatic, Amsel-positive BV for refractory and recurrent patients, or asymptomatic days in the study period for remission patients. P values were calculated using ANOVA for normally distributed data (days, age, Nugent score) and Kruskal-Wallis tests for Amsel and pH scores. Chi-square testing could not be performed on patient counts due to the low numbers of Caucasians; a Fisher’s exact test of remission versus non-remission patients had a P value of 0.02, indicating higher numbers of Caucasians in the remission group, but a confidence interval could not be assigned.

### qPCR

Vaginal swab DNA for qPCR was extracted from freshly obtained vaginal swabs, or swabs suspended in 4 ml of 2-isopropanol as described [39, 43]. This study employed the qPCR-based LbRC5 assay [39] to quantify the relative abundance of *Lactobacillus* species. This assay compares Cq values of sister wells, both amplified with broad-spectrum bacterial primers but with one well supplemented with oligomers that prevent primer binding specifically to *Lactobacillus*. The difference between these Cq values ΔCq, combined with a penalty (LbRC/5) if non-*Lactobacillus* Tm values are detected, constitutes the LbRC5 score. An LbRC5 score of 1 is generated by a population that is approximately 50% *Lactobacillus* species, and a score of 3 by a population of about 87.5% *Lactobacillus* species (1-(1/2^ΔCq^), regardless of which specific species of *Lactobacillus* is present [39, 43]. The assay was validated by correct reporting of relative abundance of species in mock mixtures of templates of vaginal species and by demonstrating effective blocking of only *Lactobacillus* species (*L*. *crispatus*, *L*. *gasseri*, *L*, *jensenii*, *L*, *iners*, *L*. *delbrueckii*, and *L*. *caseii*) among a collection of 39 other bacterial species, as described [43]. Each assay was performed with five 10-fold dilutions of an amplicon derived from *L*. *crispatus* as positive controls and 2–5 negative controls (reagents without template).

For quantitative detection of *Gardnerella* species, we designed primers that complement sequence polymorphisms in the *cpn60* gene that were specific to each of the 4 main groups identified at the study’s inception, Clades 1–4. Primers, targets, and sequences are described in S2 Table based on alignments of *cpn60* genes from 101 isolates (S1 File). We designed these in preference to the more widely used primers that target clade specific genes [33, 47, 50], since inspection of the whole genome database, larger than at the time the gene-specific primers were designed, indicated that many isolates, especially in clade 2, either have mismatches in their primer or probe binding sequences for these primers or have deletions in all or part of the target gene (S2 Fig). Their limitations include that none complement *Gsp11*, *Gsp12*, or *GS13*, represented by only 1–2 isolates in the NCBI WGS database, and that they do not differentiate *Gsp01* from *Gsp02*, *G*. *piotii* from *Gsp03*, *Gsp08* from *Gsp09* from *Gsp10*, or *G*. *swidsinskii* from *G*. *leopoldii*. In this context, our *cpn60*-based primers are remarkably consistent with groups defined as genomospecies [28]. This is to be expected, since the phylogenetic tree constructed from *cpn60* sequences from the *Gardnerella* genome sequences (S3 Fig) almost perfectly overlaps with genomospecies assignments based on whole genome comparisons [28].Primer sequences, PCR conditions, and programs are listed in S2 Table and were previously optimized and validated as described [51].

The percentage of a vaginal bacterial population of total bacteria was calculated from the molecules of the *species* relative to molecules of total bacteria, determined using the broad-spectrum primers as previously described and used also for the LbRC5 assays [42, 43]. Molecules were determined from serially diluted, sequence-verified amplicons in triplicate standard curve assays in which the amplicons were quantified fluorometrically using the QuantiFluor® ONE dsDNA System in the Quantus fluorometer (Progmega, Madison WI). qPCR assays in triplicate except that assays using primers targeting *G*. *swidsinskii/G*. *leopoldii* were only performed once due to limited sample volumes; extrapolating from triplicate runs, we estimate that at most 8% of single run reactions might under- or over-estimate abundance by ten-fold. Reactions were assembled by adding 1 μL swab DNA to 19 μL of a PCR mastermix to give final concentrations of 10 mM Tris pH 8.3, 50 mM KCl, 3 mM MgCl2, 200 μM each dNTP, 0.23 μM primer, 37.5 nM Syto 9, and were performed in a Biorad CFX Connect thermocycler. Vaginal DNA samples were determined to be free of inhibitors by confirming that they did not alter the Cq of a spiked-in control template, *Deinococcus radiodurans* 16S amplicon. Representative amplicons from all primer sets were verified by sequencing the amplicon. Under optimized cycling conditions, *cpn60* primers did not amplify templates from non-target reference species, listed in S3 Table. Cq values were rejected if the Tm value was not as predicted from the standard. Cq values that were rejected because of their Tm or because they were above the estimated limit of detection were assigned a default Cq one unit higher than the highest Cq showing the correct Tm. The geometric means of numbers of molecules per reaction were used to calculate percent molecules and used in subsequent analyses as log_10_ (%molecules).

#### Statistics

Longitudinal analyses were performed with a variety of tools, including per day assessments by ANOVA, Kaplan-Meier analysis, linear regression of the prevalence of patients above threshold abundance over time (GraphPad Prism 6.1) and mixed effect modelling. The latter used outcome groups as a factor, time as covariate, factorial analysis of both as fixed effects, random effects of patient, race and age with an optimal AR1 heterogenous model (IBM SPSS v26-27). Multinomial logistic models used restricted maximum likelihood; degrees of freedom approximated with the Satterthwaite option (IBM SPSS v26-27). Multivariate analyses and heat maps were done using tools in the online Metaboanalyst package [52–55]. Further details of specific tests are provided in figure and table legends.

## Results

### Changes in abundance of *Lactobacillus* species during and after oral metronidazole therapy

To address the timing of recovery of *Lactobacillus* species, we compared LbRC5 scores of each remission, recurrent, and refractory patient over 14 days after the initiation of oral metronidazole therapy.

**Fig 1:**
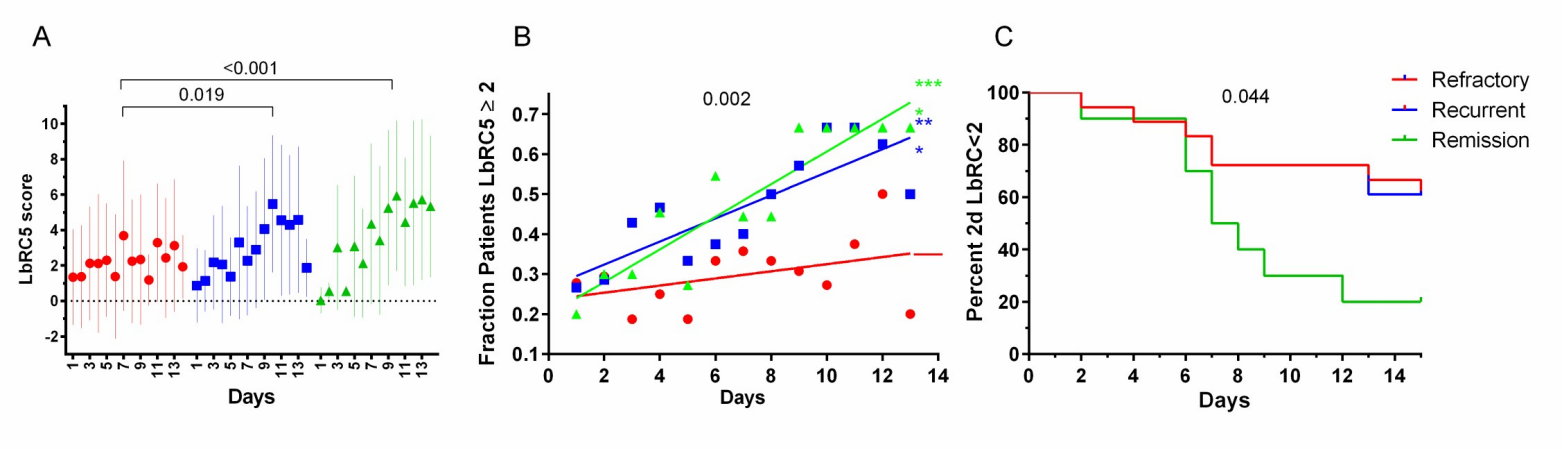
LbRC5 scores vary between clinical outcome groups. A. Mean LbRC5 scores among patients of refractory, recurrent, and remission outcome groups. Red samples = refractory patients. Blue samples = recurrent patients Green samples = remission patients. Data points are means of the log10 percent titers (= geometric means of the percents) with standard deviations. The P value was determined by mixed modeling for time dependent increases in remission versus recurrent scores. B. Fraction of patients in each clinical outcome group achieving LbRC5 scores ≥ 2 per day. Linear regression analysis (lines) indicated reasonable fits to remission and recurrent data with a common slope of 0.035, significantly different than refractory data (p = 0.002). Refractory data fit poorly to its regression line, indicating scatter (slope not significantly different from zero). C. Days from treatment initiation to LbRC5 scores >2. C. Days from initiation of treatment to sustained LbRC5 scores > 2. The first days at which LbRC5 scores were >2 for at least 2 consecutive days were recorded as hits in this Kaplan-Meier analysis; if a patient did not achieve this, she was scored as indeterminate at day 15, the day after the interval being studied. Remission scores were significantly different (p = 0.044) by log-rank analysis.

Visually, LbRC5 scores in each outcome group increased over time, remission patients increasing the most, followed by recurrent and then refractory patients (Fig 1A). Increases in LbRC5 scores over time among remission or recurrent patients compared to refractory patients were both significant and substantial (P values <0.001 and 0.019 respectively; mixed modelling with fixed effects of outcome category and time with random effects of individual patients, race and age). LbRC5 score increases over time among recurrent patients was not significantly different than remission patient score increases (p = 0.245). Scores among refractory patients did not substantially increase during this interval.

The large daily variations in individual patient LbRC5 scores resulted in large standard deviations and reflect the unstable dynamic at play in the days following treatment. These deviations rendered comparisons of daily differences between outcome groups by ANOVA or multinomial logistic analysis ineffectual. However, alternative approaches that were less sensitive to these daily fluctuations allowed comparisons of clinical outcome groups. First, the fraction of patients per outcome group on a given day that achieved LbRC5 scores ≥ 2 was lower throughout the time course among refractory patients (Fig 1B). Linear regression analysis shows that the proportion of remission and recurrent patients with above-threshold abundance increased significantly over time, whereas proportions among refractory patients did not increase.

Was there a different response among recurrent versus remission patients, not evident in the approach in Fig 1B? By scoring the time at which individual patients first began to achieve sequential days of LbRC5 scores above 2, it became clear that most remission patients achieved a more sustained and rapid LbRC5 increase (Fig 1C). Notably, sustained high *Lactobacillus* abundance varied among remission patients, some achieving this by day 2, others not until up to day 12.

In a third approach to characterizing time-dependent response to therapy, slopes of LbRC5 scores versus time from individual patients in each outcome group were determined by linear regression. Plots of these slopes (S4 Fig) showed that LbRC5 slopes of most (24 of 43) patients were significantly different from 0, but more importantly, that slopes increased significantly (Kruskal-Wallis test p = 0.037) from refractory (median 0.02, 95% CI 0.0–0.4) to recurrent (median 0.1, 95% CI 0–0.5 to remission (0.5, 95% CI 0.3–0.7) patients. The basis for the differences among outcome groups resulted from the higher proportion of patients that experienced at least some increase in LbRC5 scores over time among remission patients than refractory or recurrent patients (S4 Fig).

### *Gardnerella* Gsp07 and *G*. *swidsinskii/G*. *leopoldii* are associated with poor clinical outcome

**Fig 2:**
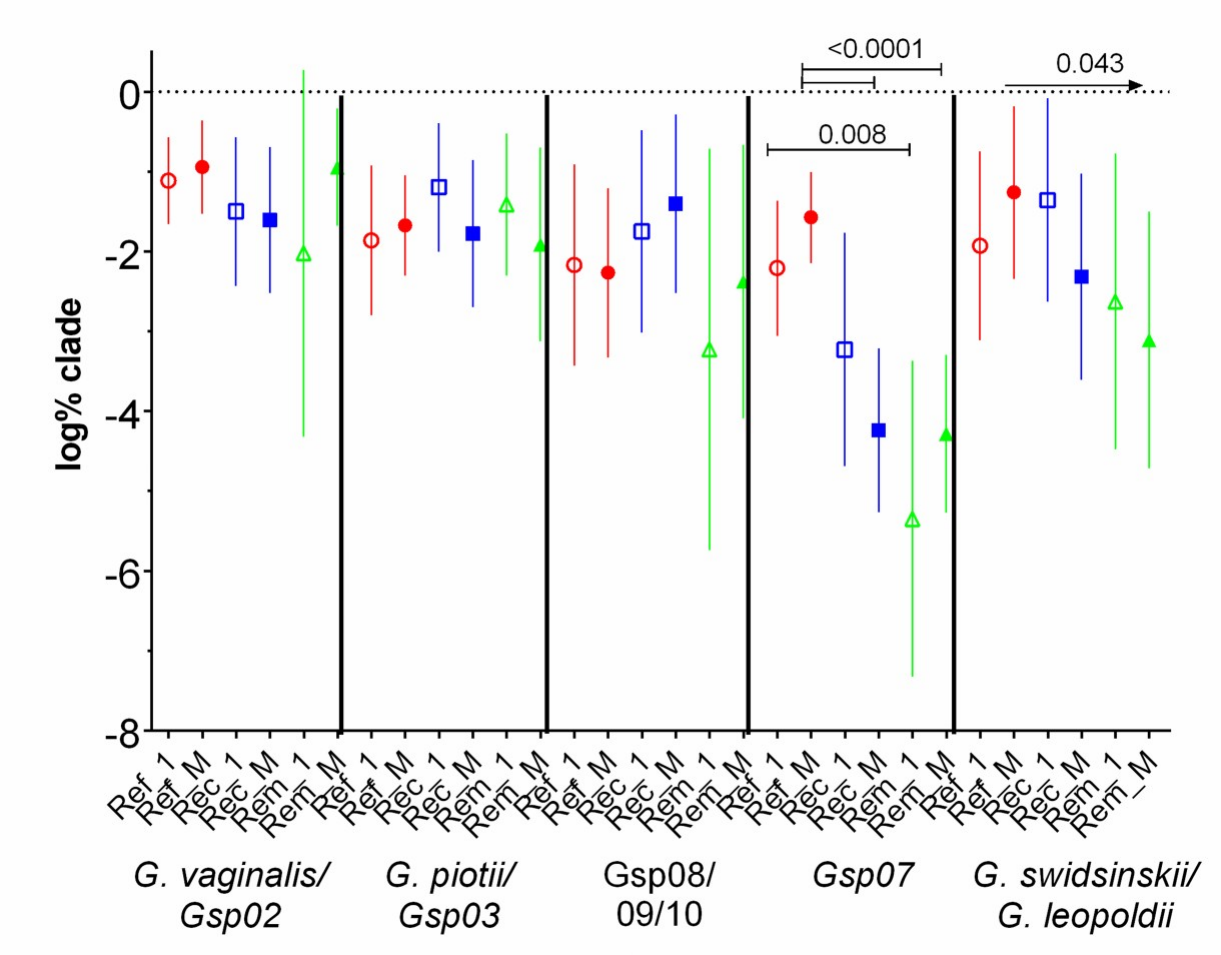
Association of abundance of *Gsp07* and *G*. *swidsinskii/G*. *leopoldii* with poor clinical response to metronidazole. Abundance of all species of *Gardnerella* were determined at enrollment (1, open symbols) and as mean abundance of days 7–14 (M, closed symbols), per patient of clades by clinical outcome group. Ref = Refractory patients, Rec = Recurrent patients, Rem = Remission patients. Means and 95% CI’s are depicted. Comparing like days between outcome groups by ANOVA post-hoc Holm-Sidak, showed significant differences among *Gsp07* abundance (only significant P values are shown); the arrow for *G*. *swidsinskii/G*. *leopoldii* comparisons indicates a significant downward trend. Paired t-tests for all 15 day 1 versus mean abundance of all clades showed no significant time-dependent changes.

In contrast to the increasing LbRC5 scores among recurrent and remission patients, abundance of each of the four species did not trend up or down in the 14 days after initiating metronidazole treatment, in any outcome group. This is seen in Fig 2 in that abundance on day 1 were not significantly different than mean abundance of all days. Most importantly, Gsp07 abundance was significantly and substantially higher among refractory patients than among recurrent or remission patients, both at day 1 and as means days 7–14 (Fig 2). Consistently, LbRC5 scores by day 14 (low abundance of *Lactobacillus* species) were negatively correlated with either high mean Gsp07 abundance (r = -0.411, 95% CI -0.634 to -0.124, p = 0.005) or to high initial *Gsp07* abundance (r = -0.456, 95% CI -0.670 to -0.171, p = 0.002). Abundance of *G*. *swidsinskii/G*. *leopoldii* isolates was associated with recurrent outcomes; these patients, who were in remission by the end of this interval, had 10-fold higher abundance than remission patients, and 10-fold lower than refractory patients. Mean abundance of *G*. *vaginalis*, *G*. *piotii/Gsp03*, and *Gsp08/Gsp09/Gsp10* were not significantly associated with clinical outcome (Fig 2). The median percentage of combined *Gardnerella* species of total bacteria at the initial, acute visit, prior to therapy, was 1.1%, ranging from 0.003% to 59.4%, and differences between outcome groups were not significant. Therefore, the specific species, *Gsp07* and *G*. *swidsinskii/G*. *leopoldii*, are associated with clinical outcome, not the total abundance of all species of *Gardnerella*.

### Association of increased numbers of species per patient with poor clinical outcome

**Fig 3:**
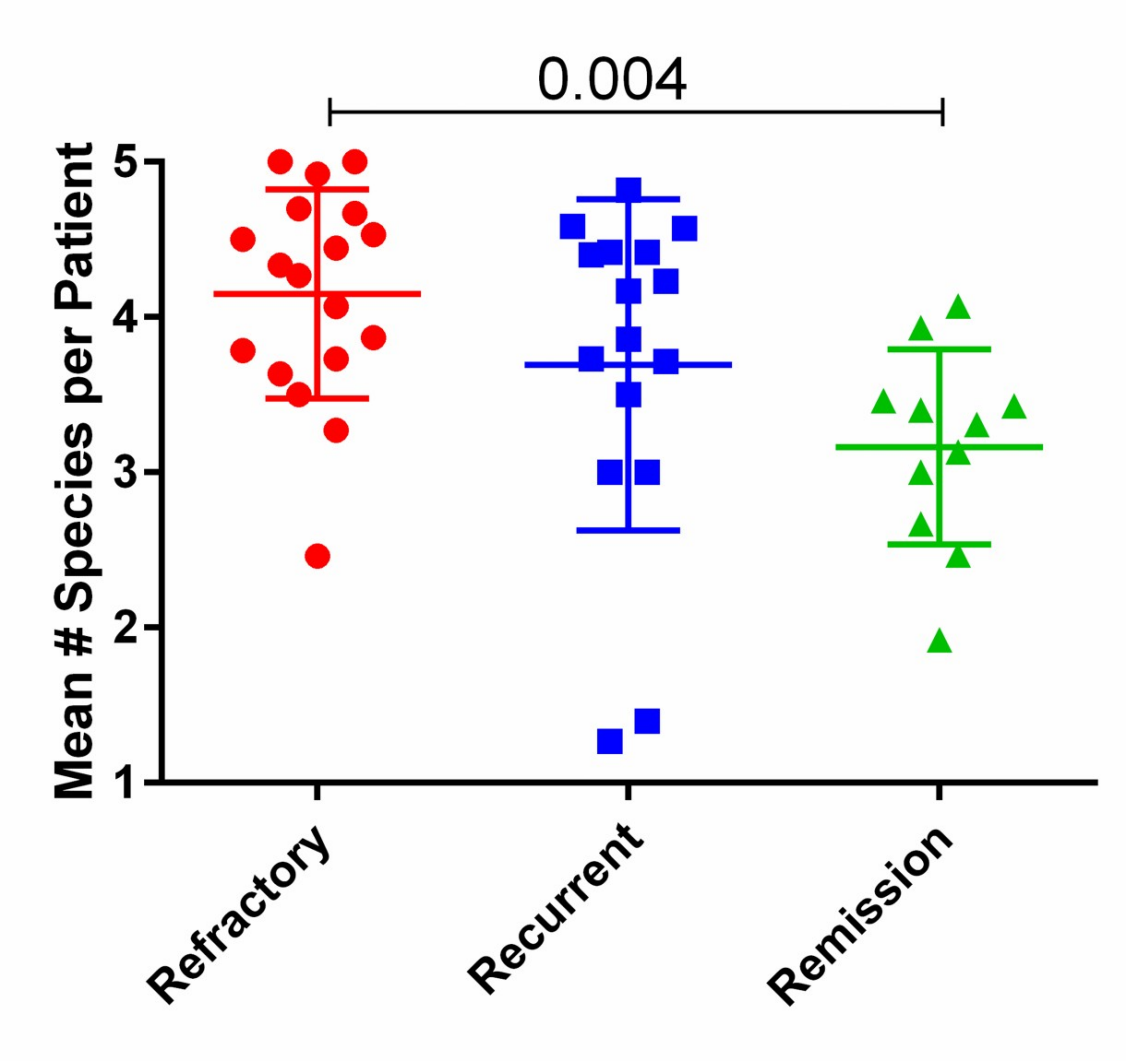
Number of *Gardnerella* species per patient. Each species was counted if present at a titer above 10x the limit of detection, then averaged for each patient over all days after initiation of oral metronidazole therapy. The p value was determined by Kruskal-Wallis test corrected for multiple comparisons.

Several studies have shown that colonization with higher numbers of clades (now species) of *Gardnerella* are associated with BV compared to non-BV patients [1, 4, 5, 15–19]. Extending this, we found that higher numbers of *Gardnerella* species, i.e., higher diversity, among BV patients were associated with clinical outcome of oral metronidazole therapy. Most refractory patients had higher numbers (4–5) of species over most days; recurrent patients had 4 species over most days; on many days, remission patients had only 2 or 3 species. These numbers did not trend up or down over time after initiation of metronidazole treatment and the following 14 days, suggesting that species were not responding differently per outcome group. This justified representing the data as the mean number of species per patient (Fig 3), which showed that refractory patients had significantly higher mean numbers compared to remission patients. Consistently, considering only the initial visit, numbers of species among refractory patients (mean 3.8, SD 1.3) was significantly higher than among remission patients (mean 2.6, SD 1.6; p = 0.022); recurrent patients were not significantly different from refractory patients at this visit (mean 3.8, SD 0.5. p = 0.062). The difference between refractory and remission patients was primarily due to the higher prevalence and abundance of *Gsp07* among refractory patients.

### Prognostic indicators of clinical outcome

Finally, we address whether any of our metrics have prognostic value of potential clinical use. We compared abundances of all species individually and in total, as well as LbRC5 scores, on the day treatment was initiated (V1), at day 7 (d7) when the 7-day regimen was complete, at d10, and at the second clinic visit (V2), typically day 14. Comparisons were made by ROC analysis, comparing non-remission (refractory and recurrent) to remission patients, refractory to non-refractory (recurrent and remission) patients, or at d10 and V2 times, recurrent to remission patients. Comparisons of significance (p ≤ 0.05) were further analyzed by Fisher’s exact tests, using the optimal threshold values from ROC curves, to calculate PPV, NPV, and Odd’s ratios. Only those comparisons with significant p values are reported (Table 3).

**Table 3:** Prognosis of clinical outcomes by relative abundance of Gsp07 or *G*. *swidsinskii/G*. *leopoldii*, and LbRC5 scores at intervals from initiation of metronidazole therapy.

Analysis	Days	V1	d7	d7	d7	d10	d10	V2	V2
	Group vs Group	Ref& Rec	Ref& Rec	Ref	Ref	Ref	Ref	Rec	Rec
		Rem	Rem	Rec& Rem	Rec& Rem	Rec& Rem	Rec& Rem	Rem	Rem
	Target	Gsp07	Gsp07	Gsp07	*Gswi/Gleo*	Gsp07	*Gswi/ Gleo*	AllGv	LbRC5
**ROC**	AUC	0.778	0.700	0.893	0.763	0.860	0.716	0.786	0.839
	95% CI	0.600	0.547	0.802	0.610	0.738	0.537	0.517	0.621
		0.956	0.852	0.984	0.916	0.981	0.895	1.055	1.057
	P	0.012	0.049	<0.0001	0.003	0.0004	0.034	0.064	0.028
	Optimal TH	-3.03	-2.05	-2.19	-2.00	-1.68	-1.14	-0.34	4.99
**Fisher’s exact**	PPV	**0.955**	**0.944**	**0.778**	0.571	**0.818**	0.625	**0.86**	**0.78**
	95% CI	0.772	0.727	0.524	0.372	0.482	0.354	0.42	0.40
		0.999	0.999	0.936	0.755	0.977	0.848	1.00	0.97
	NPV	0.381	0.385	**0.846**	**0.875**	**0.84**	**0.85**	**0.88**	**0.86**
	95% CI	0.118	0.202	0.651	0.617	0.639	0.621	0.47	0.42
		0.616	0.594	0.956	0.984	0.955	0.968	1.00	1.00
	OR	12.9	10.6	19.3	9.33	23.6	9.44	42	21
	95% CI	1.4	1.22	4.13	1.77	3.65	1.92	2.1	1.5
		116.0	92.7	89.8	49.1	153	46.4	826	293
	P	0.009	0.016	< 0.0001	0.005	0.0003	0.005	0.010	0.041

Log% abundance of each species and sums of species and of the LbRC5 scores at the indicated days were initially analyzed with ROC curves. All indicated comparison pairs were analyzed for each of these targets, only those with significant P scores are shown. The optimal threshold values (TH) were determined from the likelihood ratios and used to group individual scores as positive or negative for analysis with Fisher’s exact tests. Positive predictive values (PPV) and negative predictive values (NPV) above 0.75 are emphasized in **bold**. AUC = area under the ROC curve, approximately the test accuracy. CI = confidence interval. AllGv = log% of sums of all 5 species.

The finding of most clinical impact was that *Gsp07* above optimal thresholds at the initial visit and at day 7 predicted an eventual refractory or recurrent response, PPV 0.96 and 0.94 respectively; however, NPV values to predict remission were of no value. Day 7 and day 10 values comparing refractory to non-refractory (recurrent and remission) patients for abundance of *Gsp07* or *G*. *swidsinskii/G*. *leopoldii* had significant NPV’s > 0.84 as indicated; that is, low scores at these times predicted a non-refractory response. PPV at these times was strong for *Gsp07* abundance at day 7 and 10. Finally, at the second clinic visit, approximately 7 days after antibiotic therapy, abundance of combined species and the LbRC5 scores provided significant PPV scores, > 0.78. This indicates that by this 2^nd^ visit that high summed species abundance or low LbRC5 scores predicted eventual recurrence, and conversely that low summed species abundance or high LbRC5 scores predicted eventual remission. This LbRC5 result supports our earlier findings [39]. Abundance of species 1–3 individually did not generate significant ROC scores at any of these visits. Large confidence intervals indicate that more samples are need and/or that factors other the those considered here also contribute to patient outcome. Multivariate ROC curve analyses did not indicate that combinations of species and LbRC5 scores significantly improved predictive values above those in Table 3 for individual values, suggesting that hypothetical contributors to outcome are not among those measured in this study.

## Discussion

The most important finding of this study was that persistently high abundance of *Gardnerella Gsp07* was a signature feature of most refractory responses to oral metronidazole therapy, and that these levels did not change in a consistent manner within outcome groups, during and up to 7 days past oral metronidazole therapy. Higher abundance characteristic of refractory patients was prevalent from day 1 and allowed for predicting a refractory treatment outcome at the outset. Also important was the finding that abundance of *G*. *swidsinskii/G*. *leopoldii* was higher among recurrent patients than among remission patients, and therefore may contribute to their later recurrence. As expected, we saw increases in *Lactobacillus* abundance over the duration of therapy among remission patients; this was less pronounced in recurrent patients and not evident among refractory patients. These increases occurred after 2 to more than 12 days after the start of therapy among remission individuals. Overall, the observed associations of higher abundance of *Gsp07* and *G*. *swidsinskii/G*. *leopoldii* among poor outcome groups are not proof of causality and may only be markers of outcome. However, they also suggest a model in which higher abundance of Gsp07 or *G*. *swidsinskii/G*. *leopoldii* directly or indirectly interfere with the ability of *Lactobacillus* species to regain or maintain dominance after oral metronidazole therapy. Direct interference could result from Gsp07-specific bacteriocins; these may be encoded by some of the 129 hypothetical genes unique to Gsp07 genomes (Phylogenetic Profiler for Single Genes tool on the img.jgi.doe.gov website) [56, 57]. Indirect interference could result, for example, from synergy between *Gsp07* and other BV-associated species which are themselves antagonistic to *Lactobacillus* species.

Abundance of all species of *Gardnerella* in most patients did not decrease, and in individual cases increased, over time after initiation of metronidazole therapy. This suggests that these isolates were tolerant *in vivo* to metronidazole. In a clinical context, this is a form of resistance, but defined as tolerant in a microbiological context in the sense that species did not show overall increases in abundance over time. Moreover, the relative abundance of these species over time was not different among any clinical outcome group, suggesting that refractory or recurrent responses were not due to isolates that were more tolerant compared to isolates in the remission group. Tolerance was widespread among isolates in all outcome groups of our recurrent BV patients, likely from past exposures to therapy, suggesting that tolerance alone, while perhaps necessary, is not sufficient to determine clinical outcome.

It was recently proposed that clinical outcome of metronidazole treatment depended upon pre-treatment abundance of *L*. *iners* and *G*. *vaginalis*, and that *L*. *iners* may sequester metronidazole to then allow more growth of the initially less dominant *G*. *vaginalis* [58]. Our data do not directly support this, in that there were no significant differences between pre-treatment LbRC5 scores, relative abundance of *Gardnerella species*, or *Gardnerella species* to *Lactobacillus species* ratios, among clinical outcome groups. The only difference at the time of treatment was higher relative abundance of *Gsp07* among refractory patients. However, their concept of sequestration and/or inactivation of metronidazole is possible. We suggest that if *Gsp07* is more robust in these activities than other *Gardnerella* species, it would facilitate growth of other BV-associated species that are more sensitive to metronidazole.

Other publications used molecular approaches to study temporal dynamics of vaginal bacteria during treatment for BV [6, 50, 59, 60], but are not directly comparable to our study, since they differ one or more key metrics. Important differences include alternate cohorts by race or level of recurrence of BV, and alternate methods of identifying bacteria (species level qPCR or next-generation sequencing, or gene species-specific qPCR which excluded detection of *Gsp07)*. We note that higher levels of suppression of *G*. *vaginalis* but similar rates of resurgence of *Lactobacillus* (3–4 days) were seen by Mayer et al. [59], a study which did not enroll recurrent BV patients and most commonly used a topical metronidazole treatment.

There are limitations to this study. One is that its methodology, qPCR, limits the characterization of species to a select few, compared to the broad perspective afforded by next generation sequencing. Our *cpn60*-based primers do not detect 3 species identified by both genome-wide studies [28, 35] and do not differentiate *Gsp01* from *Gsp02*, *Gsp03* from *G*. *piotti*, or *G*. *swidsinski* from *G*. *leopoldii*. Another limitation is that LbRC5 assays do not differentiate between species of *Lactobacillus* and so do not report whether the clinical outcomes we saw were related to predominance of *L*. *crispatus* among remission patients versus *L*. *iners* among non-remission patients. This would not impact on the important correlation between *Gsp07* and *G*. *swidsinskii/G*. *leopoldii* and clinical outcome, but might define a mechanism, for example, that *Gsp07* isolates suppress *L*. *crispatus*. Further, since this approach does not provide an exhaustive profile of the vaginal microbiota, undetected species may influence clinical outcome. This limitation, nevertheless, does not detract from our observed predictive values for *Gsp07* and *G*. *swidsinskii/G*. *leopoldii*. Finally, our study is limited to a small cohort, 9–18 women, who are mostly African-American and all highly recurrent; future studies are needed to determine if conclusions from this paper will extend to large and more diverse groups.

One implication of our observations is that clinical treatment of recurrent BV may be enhanced by supplements that directly reduce abundance of *Gsp07* and *G*. *swidsinskii/G*. *leopoldii*. If these strains are sheltering in biofilm as the literature indicates [24, 25, 61, 62], then agents such as boric acid or other surfactants may be appropriate [26, 63–65]. Experimental agents are also under investigation [66]; our study suggests that investigations should document effects on *Gsp07* and *G*. *swidsinskii/G*. *leopoldii* isolates and that the most effective agents will kill these species while sparing *Lactobacillus* species.

## Supporting information

S1 Fig Genome tree of *Gardnerella* isolates from the NCBI whole genome database. The genome tree of 113 isolates was constructed from whole genome sequences by the NCBI Tree Viewer. Strain names are followed by the distance to each isolate’s nearest neighbor. Genomospecies (GS) defined by Potter et al [28] are in red; *Gardnerella* species (*Gsp*) defined by Vaneechoutte et al. [35] are in blue. Clade designations in turquoise (C#) are defined by genomic [34] and qPCR studies [37]. Distances in black at select nodes indicate summed distances between isolates in different genomospecies or *Gsp* groups. Not all isolates in this tree were included in all studies; details for each isolate are in S1 Table.
(PDF)

S2 Fig Alignment of C2 primers to Clade 2 (*G*. *piotii* & *Gsp03)* reference genome sequences. Primers were designed to the clade 2-specific hypothetical gene GI:388060098 [37]. Only the top 5 target genomes had perfect complementarity to all 3 primers; 14 genomes had multiple mismatches. Another 6 target genomes showed no alignments, so either did not encode this gene, or had gaps in their genome sequences.
(DOCX)

S3 Fig *Gardnerella cpn60*-based evolutionary analysis. Individual isolates are labelled using the Vaneechoutte et al. nomenclature [35]; nodes are labelled with the Potter et al. GS nomenclature [28], along with *cpn60* primers from this paper that target isolates included in each node (S2 Table). The evolutionary history was inferred by using the Maximum Likelihood method and Tamura-Nei model [67]. The tree with the highest log likelihood (-7151.32) is shown. The percentage of trees in which the associated taxa clustered together is shown next to the branches. Initial tree(s) for the heuristic search were obtained automatically by applying Neighbor-Join and BioNJ algorithms to a matrix of pairwise distances estimated using the Tamura-Nei model, and then selecting the topology with superior log likelihood value. The tree is drawn to scale, with branch lengths measured in the number of substitutions per site (next to the branches). This analysis involved 94 nucleotide sequences. All positions with less than 95% site coverage were eliminated, i.e., fewer than 5% alignment gaps, missing data, and ambiguous bases were allowed at any position (partial deletion option). There were a total of 1209 positions in the final dataset. Evolutionary analyses were conducted in MEGA X [68].
(DOCX)

S4 Fig Linear regression slopes of days versus *Gardnerella* species abundance or LbRC5 scores of individual patients grouped by clinical outcome. Individual slopes significantly different than 0 (0 = no change) were color coded as in Fig 1; those not different than 0 are in grey or black. Scores were calculated from 3 day moving averages (smoothed curves). The populations of slopes for each clade were not significantly different than 0 (t-tests). In contrast, slopes from LbRC5 scores of all outcome groups were significantly different from 0 and from each other (Kruskal-Wallis test, p = 0.037).
(DOCX)

S1 Table Classification of *Gardnerella* isolates into genomospecies, species, genovars, groups, or clades by genome sequence comparisons, *cpn60* sequencing/qPCR, or clade gene-specific qPCR. Nine *Gardnerella genomospecies* (GS) were classified in the 1st column, based on a consensus of 4 genome wide comparison methods [28]. In the 2nd column, 13 enumerated species of *Gardnerella*, including 3 named as *G*. *piotti*, *G*. *swidsinski*, and *G*. *leopoldii*, were assigned based on average nucleotide identity (ANI), digital DNA–DNA hybridization, and MALDI-TOF mass spectroscopy protein profiling (*Gardnerella sp1* ≡ *G*. *vaginalis*) [35]. In these two columns, * indicates an isolate not used in the studies but tentatively assigned in this table, based on their placement with author-assigned species into a common branch of the NCBI genome tree (S1 Fig). The 3rd column reports results of an older study with access to fewer isolate sequences, assigned isolates to genovars based on genome wide comparison by two neighbor-joining methods of 473 aligned core open reading frames [34]. The 4th column results are based on a signature sequence within the *cpn60* gene; almost all available isolates could be assigned to the same Gsp number [69] as the genome wide assignments [35]. Scores in this column include the original group number A-D in parentheses. These *cpn60* group letters were assigned in an earlier study and are shown in the 5th column [31]. Our study constructed a phylogenetic tree based on the entire *cpn60* open reading frame (S3 Fig) and scored them in the 6th column, using Gsp nomenclature [35]. This gene does permit differentiation some Gsp species; these are indicated by pairs of species separated by /. We also scored our *cpn60* primers (S3 Table) in column 7 based on perfect complementarity to the indicated isolates, labelled as in the previous column. Clades of *Gardnerella* as determined by the clade gene-specific (CGS) qPCR numbering system [37], are reported in the 8th column. In silico scoring of the published clade gene-specific primers or probes are based on whether they were perfectly complementary to the clade gene sequences from the NCBI genome database, versus if they had one or more mismatches (mm). Blank cells in the qPCR columns indicate that the *cpn60* sequence was not available or that the gene was not found in the genomic sequence of the isolate. CGS primers for the 4 clades targeted the following genes: *fuc1* (putative α-l-fucosidase GI:311113989; C1), *hyp* (hypothetical protein GI:388060098; C2), *thi* (thioredoxin GI:388062216; C3), *cic* (Chloride transporter GI:283783343; C4) [37].
(DOCX)

S2 Table *Gardnerella* species-specific *cpn60* primers, programs, and parameters. Primer names reflect abbreviated versus of *Gardnerella* species defined by Vaneechoutte et al. [35]. Forward (F) and Reverse R) primer sequences were manually designed from alignments of *cpn60* genes from isolates in the whole genome sequence at NCBI, checked for self and cross complementarity, compatible melts, and specificity with Primer Blast at NCBI against its Refseq representative genomes database and Nucleotide Blast against its Nucleotide collection (nr) and Whole genome shotgun contigs databases. 5’ position denotes the 5’-most position relative to the start of the open reading frame. Denaturation (Denat), annealing (Ann) and extension (Ext) temperatures are in °C, followed by seconds in parentheses; cycling followed an initial denaturation at 95°C for 60 s. Melt denotes melting temperature range monitored at the indicated time intervals in (seconds). Tm denotes melting temperature peaks in °C ± standard deviations encompassing sequence-verified amplicons and samples scored as positive. E denotes amplification efficiencies, derived from slopes of log molecule numbers versus Cq; correlation r values of linear regression curves were > 0.98. Molecule numbers of amplicons were determined by fluorescence assays using QuantiFluor® ONE dsDNA System with the Quantus fluorometer (Promega). Limits of detection (LOD) were estimated from molecules calculated to be present in standards or samples with the highest Cq values that had correct Tm values; for all primers, single molecules were detected in 5 to 32 samples (1–6% of total) so that LODs were estimated to be approximately 3 molecules per qPCR reaction, to give a reliable rate of detection based on the Poisson distribution. Samples reporting Cq values higher than this or incorrect Tm values were scored as negative and assigned a nominal number of molecules ten-fold lower than the limit of detection as upper limits. Primer specificities are demonstrated in the *cpn60* alignment fas S1 File.
(DOCX)

S3 Table Reference and clinical isolates used to validate *cpn60* primer specificity. HM numbers are references isolates obtained from BEI Resources. Clinical isolates were colony-purified from study samples and classified by sequencing their *cpn60* amplicons. *Gsp*# [35]; *GS*# [28].
(DOCX)

S1 File Alignment of 101 *Gardnerella cpn60* genes and primers. Open reading frames were extracted from the genome sequences in the NCBI database and aligned with ClustalW [69], and primers were positioned onto target aligned sequences, within Bioedit software [70]. Searches for complementary sequences to primers among non-target genes were negative. Sequences were categorized and labelled as in S1 Table.
(FAS)
