# Supplementary figures and images for: Prognosis of recurrent bacterial vaginosis based on longitudinal changes in abundance of Lactobacillus and specific species of Gardnerella
Source: PLoS One. 2021 Aug 23;16(8):e0256445. doi: 10.1371/journal.pone.0256445 (PMC8382169; doi:10.1371/journal.pone.0256445)

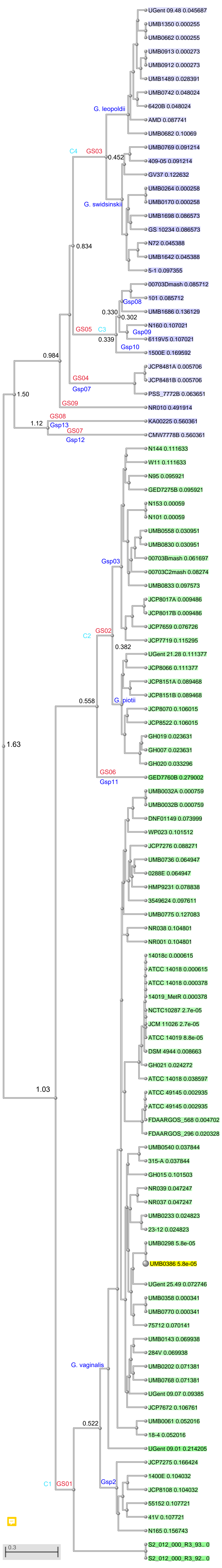

Supplement: S1 Fig — The genome tree of 113 isolates was constructed from whole genome sequences by the NCBI Tree Viewer. Strain names are followed by the distance to each isolate’s nearest neighbor. Genomospecies (GS) defined by Potter et al [28] are in red; Gardnerella species (Gsp) defined by Vaneechoutte et al. [35] are in blue. Clade designations in turquoise (C#) are defined by genomic [34] and qPCR studies [37]. Distances in black at select nodes indicate summed distances between isolates in different genomospecies or Gsp groups. Not all isolates in this tree were included in all studies; details for each isolate are in S1 Table. (PDF) [file pone.0256445.s001.pdf]
