# Supplementary material for: Prognosis of recurrent bacterial vaginosis based on longitudinal changes in abundance of Lactobacillus and specific species of Gardnerella
Source: PLoS One. 2021 Aug 23;16(8):e0256445. doi: 10.1371/journal.pone.0256445 (PMC8382169; doi:10.1371/journal.pone.0256445)
Supplement: S2 Fig — Primers were designed to the clade 2-specific hypothetical gene GI:388060098 [37]. Only the top 5 target genomes had perfect complementarity to all 3 primers; 14 genomes had multiple mismatches. Another 6 target genomes showed no alignments, so either did not encode this gene, or had gaps in their genome sequences. (DOCX) [file pone.0256445.s002.docx]

10 20 30 40 50 60 70 80 90 100 110 120 130

....|....|....|....|....|....|....|....|....|....|....|....|....|....|....|....|....|....|....|....|....|....|....|....|....|....|

Gv2_hyp_S (Sense) **---GCAAAGCAGACTGAGCGTATTAG--------------------------------------------------------------------------------------------------------**

Gv2_hyp_AS (Antisense) **--------------------------------------------------------------------------------------------------------GCGATGAGGAGCCTGATTATTAC---**

Gv2_hyp_TM(Antisense) **-------------------------------------------------------------------~--TGCACTGTTATGCGAGCGCCTGCG------------------------------------**

Gvag UMB0830 .21837_8_82. **TTTGCAAAGCAGACTGAGCGTATTAGAAAAGCTCAAAAAAGTTAATCCAAATATGACTTTATTTGTTTTTTGCACTGTTATGCGAGCGCCTGCGTATAACAGCAGCGATGAGGAGCCTGATTATTACGCA**

Gvag JCP7719 HMPREF1576-1 **TTTGCAAAGCAGACTGAGCGTATTAGAAAAGCTCAAAAAAGTTAATCCAAATATGACTTTATTTGTTTTTTGCACTGTTATGCGAGCGCCTGCGTATAACAGCAGCGATGAGGAGCCTGATTATTACGCA**

Gvag JCP8017A HMPREF1577- **TTTGCAAAGCAGACTGAGCGTATTAGAAAAGCTCAAAAAAGTTAATCCAAATATGACTTTATTTGTTTTTTGCACTGTTATGCGAGCGCCTGCGTATAACAGCAGCGATGAGGAGCCTGATTATTACGCA**

Gvag JCP8017B HMPREF1578- **TTTGCAAAGCAGACTGAGCGTATTAGAAAAGCTCAAAAAAGTTAATCCAAATATGACTTTATTTGTTTTTTGCACTGTTATGCGAGCGCCTGCGTATAACAGCAGCGATGAGGAGCCTGATTATTACGCA**

Gvag 00703Bmash ctg00017, **TTTGCAAAGCAGACTGAGCGTATTAGAAAAGCTCAAAAAAGTTAATCCAAATATGACTTTATTTGTTTTTTGCACTGTTATGCGAGCGCCTGCGTATAACAGCAGCGATGAGGAGCCTGATTATTACGCA**

Gvag UMB0833 .21837_8_90. **TTTGCAAAGCAGACTGAGCGTATTGGAAAAGCTCAAAAAAGTTAATCCAAATATGACTTTATTTGTTTTTTGCACTGTTATGCGCGCGCCTGCGTATAACAGCAGCGATGAGGAGCCTGATTATTACGCA**

Gvag JCP8151B HMPREF1583- **TTTGCAAAGCAGACTTAGCGTATTGGAAAAGCTCAAAAAAGTTAATCCAAATATGACTTTATTTGTTTTTTGCACTGTTATGCGAGCGCCTGCGTATAACAGCAGCGATGAGGAGCCTGATTATTACGCA**

Gvag JCP8522 HMPREF1586-1 **TTTGCAAAGCAGACTGAGCGTATTGGAAAAGCTCAAAAAAGTTAATCCAAATATGACTTTATTTGTTTTTTGCACTGTTATGCGCGCGCCTGCGTATAACAGCAGCGATGAGGAGCCTGATTATTACGCA**

Gvag W11 2556926469, LGOX **TTTGCAAAGCAGACTGAGCGTATTGGAAAAGCTCAAAAAAGTTAATCCAAATATGACTTTATTTGTTTTTTGCACTGTTATGCGCGCGCCTGCGTATAACAGCAGCGATGAGGAGCCTGATTATTACGCA**

Gvag JCP8066 HMPREF1579-1 **TTTGCAAAGTCGCCTGCGCGTATTAGAAAAGCTCAAAAAAGTTAATCCAAATATGACTTTATTTGTTTTTTGCACTGTTATGCGCGCGCCTGCGTATAACAGCAGCGATGAGGAGCCTGATTATTACGCA**

Gvag JCP8070 HMPREF1580-1 **TTTGCAAAGTCGCCTGCGCGTATTAGAAAAGCTCAAAAAAGTTAATCCAAATATGACTTTATTTGTTTTTTGCACTGTTATGCGCGCGCCTGCGTATAACAGCAGCGATGAGGAGCCTGATTATTACGCA**

Gvag UGent 21.28 VMF2128C **TTTGCAAAGTCGCCTGCGCGTATTAGAAAAGCTCAAAAAAGTTAATCCAAATATGACTTTATTTGTTTTTTGCACTGTTATGCGCGCGCCTGCGTATAACAGCAGCGATGAGGAGCCTGATTATTACGCA**

Gvag JCP8151A HMPREF1582- **TTTGCAAAGTCGCCTGCGCGTATTAGAAAAGCTCAAAAAAGTTAATCCAAATATGACTTTATTTGTTTTTTGCACTGTTATGCGCGCGCCTGCGTATAACAGCAGCGATGAGGAGCCTGATTATTACGCA**

Gvag UGent 18.01 NODE_1_l **TTTGCAAAGTCGCCTGCGCGTATTAGAAAAGCTCAAAAAAGTTAATCCAAATATGACTTTATTTGTTTTTTGCACTGTTATGCGCGCGCCTGCGTATAACAGCAGCGATGAGGAGCCTGATTATTACGCA**

Gvag GH019 scaffold1, NNR **TTTGCAAAGTCGCCTGCGCGTATTAGAAAAGCTCAAAAAAGTTAATCCAAATATGACTTTATTTGTTTTTTGCACTGTTATGCGCGCGCCTGCGTATAACAGCAGCGATGAGGAGCCTGATTATTACGCA**

Gvag JCP7659 HMPREF1574-1 **TTTGCAAAGTCGCCTGAACGTATTGGAAAAGCTCAAAAAAGTTAATCCAAATATGACTTTATTTGTTTTTTGCACTGTTATGCGAGCGCCTGCGTATAACAGCAGCGATGAGGAGCCTGATTATTACGCA**

Gvag N144 scaffold1, LRTT **TTTGCAAAGTCGCCTGAACGTATTAGAAAAGCTCAAAAAAGTTAATCCAAATATGACTTTATTTGTTTTTTGCACTGTTATGCGAGCGCCTGCGTATAACAGCAGCGATGAGGAGCCTGATTATTACGCA**

Gvag GH020 scaffold1, NNR **TTTGCAAAGTCGCCTGCGCGTATTAGAAAAGCTCAAAAAAGTTAATCCAAATATGACTTTATTTGTTTTTTGCACTGTTATGCGCGCGCCTGCGTATAACAGCAGCGATGAGGAGCCTGATTATTACGCA**

Gvag GH007 scaffold3, NNR **TTTGCAAAGTCGCCTGCGCGTATTAGAAAAGCTCAAAAAAGTTAATCCAAATATGACTTTATTTGTTTTTTGCACTGTTATGCGCGCGCCTGCGTATAACAGCAGCGATGAGGAGCCTGATTATTACGCA**

**S2 Fig**. **Alignment of C2 primers to Clade 2 (*G. piotii* & *Gsp03)* reference genome sequences**. Primers were designed to the clade 2-specific hypothetical gene GI:388060098 [37] . Only the top 5 target genomes had perfect complementarity to all 3 primers; 14 genomes had multiple mismatches. Another 6 target genomes showed no alignments, so either did not encode this gene, or had gaps in their genome sequences.

.
