# Supplementary material for: Prognosis of recurrent bacterial vaginosis based on longitudinal changes in abundance of Lactobacillus and specific species of Gardnerella
Source: PLoS One. 2021 Aug 23;16(8):e0256445. doi: 10.1371/journal.pone.0256445 (PMC8382169; doi:10.1371/journal.pone.0256445)
Supplement: S3 Fig — Individual isolates are labelled using the Vaneechoutte et al. nomenclature [35]; nodes are labelled with the Potter et al. GS nomenclature [28], along with cpn60 primers from this paper that target isolates included in each node (S2 Table). The evolutionary history was inferred by using the Maximum Likelihood method and Tamura-Nei model [67]. The tree with the highest log likelihood (-7151.32) is shown. The percentage of trees in which the associated taxa clustered together is shown next to the branches. Initial tree(s) for the heuristic search were obtained automatically by applying Neighbor-Join and BioNJ algorithms to a matrix of pairwise distances estimated using the Tamura-Nei model, and then selecting the topology with superior log likelihood value. The tree is drawn to scale, with branch lengths measured in the number of substitutions per site (next to the branches). This analysis involved 94 nucleotide sequences. All positions with less than 95% site coverage were eliminated, i.e., fewer than 5% alignment gaps, missing data, and ambiguous bases were allowed at any position (partial deletion option). There were a total of 1209 positions in the final dataset. Evolutionary analyses were conducted in MEGA X [68]. (DOCX) [file pone.0256445.s003.docx]

**
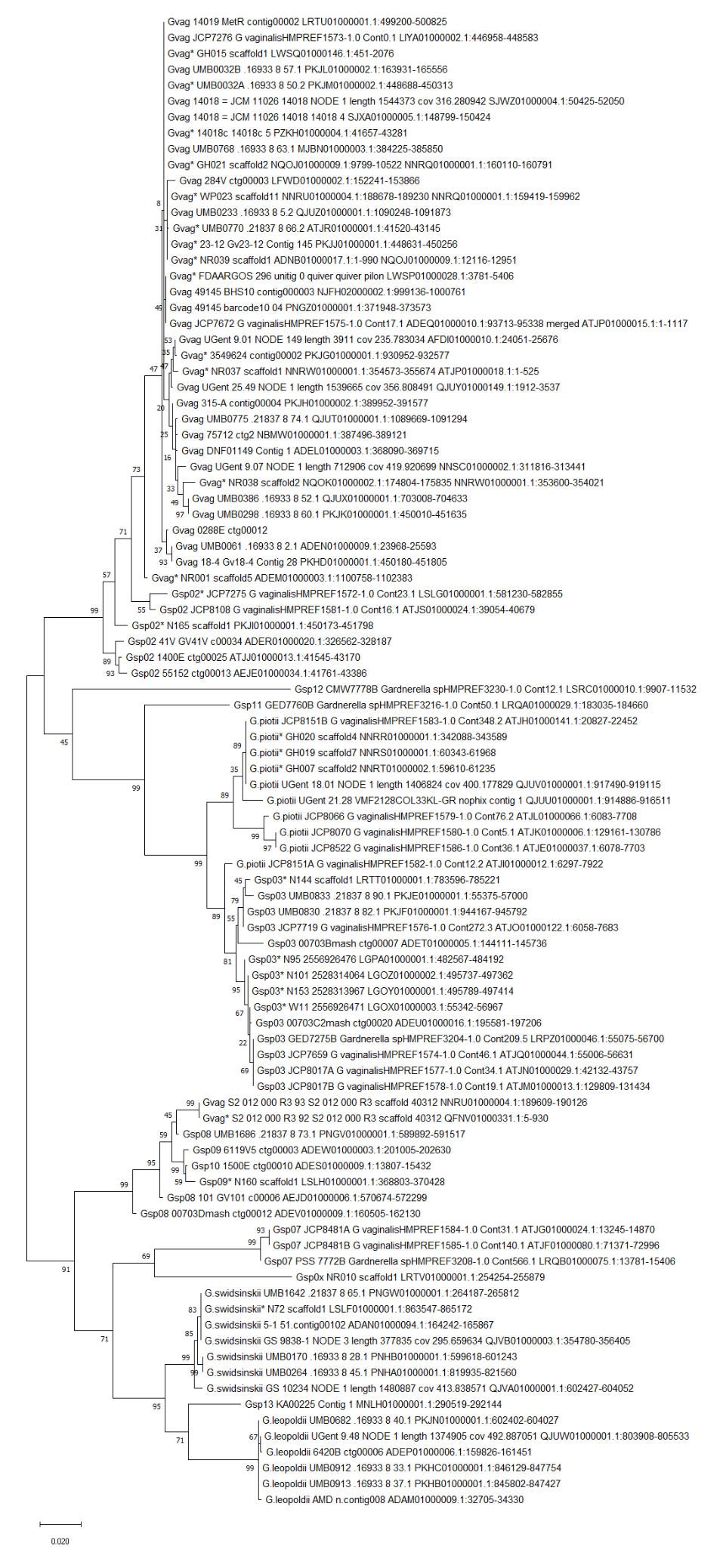
**

*GS01 Gvag.Gsp02 cpn60*

*GS02 Gpio.Gsp03 cpn60*

*GS03 Gswi.Gleo cpn60*

*GS05 Gsp08-10 cpn60*

**S3 Fig. Gardnerella *cpn60*-based evolutionary analysis.**

Individual isolates are labelled using the Vaneechoutte et al. nomenclature [35]; nodes are labelled with the Potter et al. GS nomenclature [28], along with Cpn60 primers from this paper that target isolates included in each node (S2 Table). The evolutionary history was inferred by using the Maximum Likelihood method and Tamura-Nei model [67]. The tree with the highest log likelihood (-7151.32) is shown. The percentage of trees in which the associated taxa clustered together is shown next to the branches. Initial tree(s) for the heuristic search were obtained automatically by applying Neighbor-Join and BioNJ algorithms to a matrix of pairwise distances estimated using the Tamura-Nei model, and then selecting the topology with superior log likelihood value. The tree is drawn to scale, with branch lengths measured in the number of substitutions per site (next to the branches). This analysis involved 94 nucleotide sequences. All positions with less than 95% site coverage were eliminated, i.e., fewer than 5% alignment gaps, missing data, and ambiguous bases were allowed at any position (partial deletion option). There were a total of 1209 positions in the final dataset. Evolutionary analyses were conducted in MEGA X [68].

*GS11*

*GS08*

*GS07*

*GS09*

*GS04 Gsp07 cpn60*
