# Supplementary material for: Prognosis of recurrent bacterial vaginosis based on longitudinal changes in abundance of Lactobacillus and specific species of Gardnerella
Source: PLoS One. 2021 Aug 23;16(8):e0256445. doi: 10.1371/journal.pone.0256445 (PMC8382169; doi:10.1371/journal.pone.0256445)
Supplement: S4 Fig — Individual slopes significantly different than 0 (0 = no change) were color coded as in Fig 1; those not different than 0 are in grey or black. Scores were calculated from 3 day moving averages (smoothed curves). The populations of slopes for each clade were not significantly different than 0 (t-tests). In contrast, slopes from LbRC5 scores of all outcome groups were significantly different from 0 and from each other (Kruskal-Wallis test, p = 0.037). (DOCX) [file pone.0256445.s004.docx]

**S4 Fig.** **Linear regression slopes of days versus *Gardnerella* species abundance or LbRC5 scores of individual patients grouped by clinical outcome**.

Individual slopes significantly different than 0 (0 = no change) were color coded as in Fig. 1; those not different than 0 are in grey or black. Scores were calculated from 3 day moving averages (smoothed curves). The populations of slopes for each clade were not significantly different than 0 (t-tests). In contrast, slopes from LbRC5 scores of all outcome groups were significantly different from 0 and from each other (Kruskal-Wallis test, p = 0.037).
