# Supplementary material for: Prognosis of recurrent bacterial vaginosis based on longitudinal changes in abundance of Lactobacillus and specific species of Gardnerella
Source: PLoS One. 2021 Aug 23;16(8):e0256445. doi: 10.1371/journal.pone.0256445 (PMC8382169; doi:10.1371/journal.pone.0256445)
Supplement: S1 Table — Nine Gardnerella genomospecies (GS) were classified in the 1st column, based on a consensus of 4 genome wide comparison methods [28]. In the 2nd column, 13 enumerated species of Gardnerella, including 3 named as G. piotti, G. swidsinski, and G. leopoldii, were assigned based on average nucleotide identity (ANI), digital DNA–DNA hybridization, and MALDI-TOF mass spectroscopy protein profiling (Gardnerella sp1 ≡ G. vaginalis) [35]. In these two columns, * indicates an isolate not used in the studies but tentatively assigned in this table, based on their placement with author-assigned species into a common branch of the NCBI genome tree (S1 Fig). The 3rd column reports results of an older study with access to fewer isolate sequences, assigned isolates to genovars based on genome wide comparison by two neighbor-joining methods of 473 aligned core open reading frames [34]. The 4th column results are based on a signature sequence within the cpn60 gene; almost all available isolates could be assigned to the same Gsp number [69] as the genome wide assignments [35]. Scores in this column include the original group number A-D in parentheses. These cpn60 group letters were assigned in an earlier study and are shown in the 5th column [31]. Our study constructed a phylogenetic tree based on the entire cpn60 open reading frame (S3 Fig) and scored them in the 6th column, using Gsp nomenclature [35]. This gene does permit differentiation some Gsp species; these are indicated by pairs of species separated by /. We also scored our cpn60 primers (S3 Table) in column 7 based on perfect complementarity to the indicated isolates, labelled as in the previous column. Clades of Gardnerella as determined by the clade gene-specific (CGS) qPCR numbering system [37], are reported in the 8th column. In silico scoring of the published clade gene-specific primers or probes are based on whether they were perfectly complementary to the clade gene sequences from the NCBI genome database, versus if th [file pone.0256445.s005.docx]

| S1 Table. Classification of *Gardnerella* isolates into genomospecies, species, genovars, groups, or clades by genome sequencing*, cpn60* sequencing/qPCR, or clade gene-specific qPCR | | | | | | | | | | |  |  |  |  |
| --- | --- | --- | --- | --- | --- | --- | --- | --- | --- | --- | --- | --- | --- | --- |
| Method: | **Genome sequence** | | | | ***cpn60* sequence** | | | | ***cpn60* qPCR** | **CGS qPCR** |  |  |  |  |
| Isolate | GS  [1] | G. sp.  [2] | Genovar  [3] | | G. sp.  [4] | | Group  [5] | S3 Fig | S1 File | Clade [6] |  |  | C1 | C1 |
| 75712 | GS01 | 1 | | 1 | 1(C) | | C | 1/2 | 1/2 | C1 |  |  |  |  |
| 0288E | GS01 | 1 | | 1 | 1(C) | | C | 1/2 | 1/2 | C1 |  |  |  |  |
| 18-4 | GS01 | 1 | |  |  | |  | 1/2 | 1/2 | C1 |  |  |  |  |
| 284V | GS01 | 1 | | 1 | 1(C) | | C | 1/2 | 1/2 | C1 |  |  |  |  |
| 315-A | GS01 | 1 | |  | 1(C) | | C | 1/2 | 1/2 | C1 |  |  |  |  |
| ATCC 14018 | GS01 | 1 | | 1 | 1(C) | | C |  | 1/2 | mm |  |  |  |  |
| ATCC 14019 | GS01 | 1 | | 1 | 1(C) | | C |  | 1/2 | mm |  |  |  |  |
| ATCC 49145 | GS01 | 1 | |  | 1(C) | | C |  | 1/2 | C1 |  |  |  |  |
| DNF01149 | GS01 | 1 | |  |  | |  | 1/2 | 1/2 | C1 |  |  |  |  |
| JCP7276 | GS01 | 1 | |  | 1(C) | | C | 1/2 | 1/2 | C1 |  |  |  |  |
| JCP7672 | GS01 | 1 | |  | 1(C) | | C | 1/2 | 1/2 | C1 |  |  |  |  |
| S2_012_000_R3_93 | GS01 | 1 | |  |  | |  |  | 8/9/10 | C1 |  |  |  |  |
| UGent 09.01 | GS01 | 1 | |  | 1(C) | | C |  | 1/2 | mm |  |  |  |  |
| UGent 09.07 | GS01 | 1 | |  | 1(C) | | C |  | 1/2 | C1 |  |  |  |  |
| UGent 25.49 | GS01 | 1 | |  | 1(C) | | C | 1/2 | 1/2 | C1 |  |  |  |  |
| UMB0032B | GS01 | 1 | |  |  | |  | 1/2 | 1/2 | C1 |  |  |  |  |
| UMB0061 | GS01 | 1 | |  |  | |  | 1/2 | 1/2 | C1 |  |  |  |  |
| UMB0233 | GS01 | 1 | |  |  | |  | 1/2 | 1/2 | C1 |  |  |  |  |
| UMB0298 | GS01 | 1 | |  |  | |  | 1/2 | 1/2 | C1 |  |  |  |  |
| UMB0386 | GS01 | 1 | |  |  | |  | 1/2 | 1/2 | C1 |  |  |  |  |
| UMB0775 | GS01 | 1 | |  |  | |  | 1/2 | 1/2 | C1 |  |  |  |  |
| 3549624 | GS01 | 1* | |  | 1(C) | | C | 1/2 | 1/2 | C1 |  |  |  |  |
| 23-12 | GS01 | 1* | |  |  | |  | 1/2 | 1/2 | C1 |  |  |  |  |
| FDAARGOS_296 | GS01 | 1* | |  |  | |  | 1/2 | 1/2 | C1 |  |  |  |  |
| GH015 | GS01 | 1* | |  |  | |  | 1/2 | 1/2 | C1 |  |  |  |  |
| GH021 | GS01 | 1* | |  |  | |  | 1/2 | 1/2 | C1 |  |  |  |  |
| HMP9231 | GS01 | 1* | |  | 1(C) | | C |  | 1/2 | mm |  |  |  |  |
| JCM 11026 | GS01 | 1* | |  |  | |  |  | 1/2 | mm |  |  |  |  |
| NR001 | GS01 | 1* | |  |  | |  | 1/2 | 1/2 | C1 |  |  |  |  |
| NR037 | GS01 | 1* | |  |  | |  | 1/2 | 1/2 | C1 |  |  |  |  |
| NR038 | GS01 | 1* | |  |  | |  | 1/2 | 1/2 | C1 |  |  |  |  |
| NR039 | GS01 | 1* | |  |  | |  | 1/2 | 1/2 | C1 |  |  |  |  |
| S2_012_000_R3_92 | GS01 | 1* | |  |  | |  |  | 8/9/10 | C1 |  |  |  |  |
| UMB0032A | GS01 | 1* | |  |  | |  | 1/2 | 1/2 | C1 |  |  |  |  |
| UMB0358 | GS01 | 1* | |  |  | |  |  | 1/2 | C1 |  |  |  |  |
| UMB0770 | GS01 | 1* | |  |  | |  | 1/2 | 1/2 | C1 |  |  |  |  |
| WP023 | GS01 | 1* | |  |  | |  | 1/2 | 1/2 | C1 |  |  |  |  |
| DSM 4944 | GS01* | 1* | |  |  | |  |  |  |  |  |  |  |  |
| Method: | **Genome sequence** | | | | **Cpn60 sequence** | | | | **Cpn60 qPCR** | **CGS qPCR** |  |  |  |  |
| Isolate | GS  [1] | G. sp.  [2] | Genovar  [3] | | G. sp.  [4] | Group  [5] | | S3 Fig | S1 File | Clade [6] |  |  | C1 | C1 |
| FDAARGOS_568 | GS01* | 1* | |  |  |  | |  | 1/2 |  |  |  |  |  |
| NCTC10287 | GS01* | 1* | |  |  |  | |  | 1/2 |  |  |  |  |  |
| UMB0143 | GS01* | 1* | |  |  |  | |  | 1/2 | C1 |  |  |  |  |
| UMB0202 | GS01* | 1* | |  |  |  | |  | 1/2 | C1 |  |  |  |  |
| UMB0540 | GS01* | 1* | |  |  |  | |  | 1/2 | C1 |  |  |  |  |
| UMB0736 | GS01* | 1* | |  |  |  | |  | 1/2 | C1 |  |  |  |  |
| 55152 | GS01 | 2 | | 1 | 2(C) |  | | 1/2 | 1/2 | mm |  |  |  |  |
| 1400E | GS01 | 2 | | 1 | 2(C) |  | | 1/2 | 1/2 | C1 |  |  |  |  |
| 41V | GS01 | 2 | |  | 2(C) | C | | 1/2 | 1/2 | mm |  |  |  |  |
| JCP8108 | GS01 | 2 | |  | 1(C) |  | | 1/2 | 1/2 | mm |  |  |  |  |
| JCP7275 | GS01 | 2* | |  | 1(C) | C | | 1/2 | 1/2 | mm |  |  |  |  |
| N165 | GS01 | 2* | |  |  | C | | 1/2 | 1/2 | mm |  |  |  |  |
| 00703Bmash | GS02 | 3 | | 2 |  |  | | 3/Gpio | 3/Gpio | C2 |  |  |  |  |
| 00703C2mash | GS02 | 3 | | 2 | 3(B) | B | | 3/Gpio | 3/Gpio | nd |  |  |  |  |
| GED7275B | GS02 | 3 | |  | 3(B) | B | | 3/Gpio | 3/Gpio | nd |  |  |  |  |
| JCP7659 | GS02 | 3 | |  | 3(B) | B | | 3/Gpio | 3/Gpio | mm |  |  |  |  |
| JCP7719 | GS02 | 3 | |  | 3(B) |  | | 3/Gpio | 3/Gpio | C2 |  |  |  |  |
| JCP8017A | GS02 | 3 | |  | 3(B) | B | | 3/Gpio | 3/Gpio | C2 |  |  |  |  |
| JCP8017B | GS02 | 3 | |  | 3(B) | B | | 3/Gpio | 3/Gpio | C2 |  |  |  |  |
| UMB0830 | GS02 | 3 | |  |  |  | | 3/Gpio | 3/Gpio | C2 |  |  |  |  |
| UMB0833 | GS02 | 3 | |  |  |  | | 3/Gpio | 3/Gpio | mm |  |  |  |  |
| N101 | GS02 | 3* | |  |  | B | | 3/Gpio | 3/Gpio | nd |  |  |  |  |
| N144 | GS02 | 3* | |  |  | B | | 3/Gpio | 3/Gpio | mm |  |  |  |  |
| N153 | GS02 | 3* | |  |  | B | | 3/Gpio | 3/Gpio | nd |  |  |  |  |
| N95 | GS02 | 3* | |  |  | B | | 3/Gpio | 3/Gpio | nd |  |  |  |  |
| W11 | GS02 | 3* | |  |  | B | | 3/Gpio | 3/Gpio | mm |  |  |  |  |
| UMB0558 | GS02* | 3* | |  |  |  | |  | 3/Gpio | C2 |  |  |  |  |
| UGent 21.28 | GS02 | G. piotii | |  | 4(B) | B | | 3/Gpio | 3/Gpio | mm |  |  |  |  |
| UGENT 18.01 | GS02 | G. piotti | |  | 4(B) | B | | 3/Gpio | 3/Gpio | mm |  |  |  |  |
| JCP8066 | GS02 | G.piotii | |  | 4(B) | B | | 3/Gpio | 3/Gpio | mm |  |  |  |  |
| JCP8070 | GS02 | G.piotii | |  | 4(B) | B | | 3/Gpio | 3/Gpio | mm |  |  |  |  |
| JCP8151A | GS02 | G.piotii | |  | 4/3(B) | B | | 3/Gpio | 3/Gpio | mm |  |  |  |  |
| JCP8151B | GS02 | G.piotii | |  | 4(B) | B | | 3/Gpio | 3/Gpio | mm |  |  |  |  |
| JCP8522 | GS02 | G.piotii | |  | 4(B) | B | | 3/Gpio | 3/Gpio | mm |  |  |  |  |
| GH007 | GS02 | G.piotii* | |  |  |  | | 3/Gpio | 3/Gpio | mm |  |  |  |  |
| GH020 | GS02 | G.piotii* | |  |  |  | | 3/Gpio | 3/Gpio | mm |  |  |  |  |
| GH019 | GS02* | G.piotii* | |  |  |  | | 3/Gpio | 3/Gpio |  |  |  |  |  |
| 5-1 | GS03 | G. swidsinskii | | 4 | 6(A) | A | | Gswi/Gleo | Gswi/Gleo | C4 |  |  |  |  |
| 9838-1 | GS03 | G. swidsinskii | |  | 6(A) | A | |  | Gswi/Gleo |  |  |  |  |  |
| GS 10234 | GS03 | G. swidsinskii | |  | 6(A) | A | | Gswi/Gleo | Gswi/Gleo | C4 |  |  |  |  |
| Method: | **Genome sequence** | | | | **Cpn60 sequence** | | | | **Cpn60 qPCR** | **CGS qPCR** |  |  |  |  |
| Isolate | GS  [1] | G. sp.  [2] | Genovar  [3] | | G. sp.  [4] | Group  [5] | | S3 Fig | S1 File | Clade [6] |  |  |  |  |
| UMB0170 | GS03 | G. swidsinskii | |  |  |  | | Gswi/Gleo |  | C4 |  |  |  |  |
| UMB0264 | GS03 | G. swidsinskii | |  |  |  | | Gswi/Gleo |  | C4 |  |  |  |  |
| UMB1642 | GS03 | G. swidsinskii | |  |  |  | | Gswi/Gleo | Gswi/Gleo | C4 |  |  |  |  |
| 409-05 | GS03 | G. swidsinskii* | | 4 | 6(A) | A | |  |  | C4 |  |  |  |  |
| GV37 | GS03 | G. swidsinskii* | |  | 6(A) | A | |  |  | C4 |  |  |  |  |
| N72 | GS03 | G. swidsinskii* | |  |  |  | | Gswi/Gleo | Gswi/Gleo | C4 |  |  |  |  |
| UMB0769 | GS03* | G. swidsinskii* | |  |  |  | |  | Gswi/Gleo |  |  |  |  |  |
| UMB1698 | GS03* | G. swidsinskii* | |  |  |  | |  | Gswi/Gleo | C4 |  |  |  |  |
| UGENT 06.41 | GS03 | G. leopoldii | |  | 5(A) | A | |  |  | nd |  |  |  |  |
| UGent 09.48 | GS03 | G. leopoldii | |  | 5(A) | A | |  | Gswi/Gleo | C4 |  |  |  |  |
| 6420B | GS03 | G.leopoldii | | 4 | 5(A) | A | | Gswi/Gleo | Gswi/Gleo | mm |  |  |  |  |
| AMD | GS03 | G.leopoldii | | 4 | 5(A) | A | | Gswi/Gleo | Gswi/Gleo | C4 |  |  |  |  |
| UMB0682 | GS03 | G.leopoldii | |  |  |  | | Gswi/Gleo | Gswi/Gleo | C4 |  |  |  |  |
| UMB0913 | GS03 | G.leopoldii | |  |  |  | | Gswi/Gleo | Gswi/Gleo | mm |  |  |  |  |
| UMB0912 | GS03* | G.leopoldii | |  |  |  | | Gswi/Gleo | Gswi/Gleo |  |  |  |  |  |
| UMB0662 | GS03* | G.leopoldii* | |  |  |  | |  | Gswi/Gleo |  |  |  |  |  |
| UMB0742 | GS03* | G.leopoldii* | |  |  |  | |  | Gswi/Gleo | C4 |  |  |  |  |
| UMB1350 | GS03* | G.leopoldii* | |  |  |  | |  | Gswi/Gleo |  |  |  |  |  |
| UMB1489 | GS03* | G.leopoldii* | |  |  |  | |  | Gswi/Gleo |  |  |  |  |  |
| 6420LIT |  | G.leopoldii | | 4 |  |  | |  |  |  |  |  |  |  |
| JCP8481A | GS04 | 7 | |  | 7 |  | | 7 | 7 |  |  |  |  |  |
| JCP8481B | GS04 | 7 | |  | 7 |  | | 7 | 7 |  |  |  |  |  |
| PSS_7772B | GS04 | 7 | |  | 7 |  | | 7 | 7 |  |  |  |  |  |
| 101 | GS05 | 8 | |  | 8b(D) | D | | 8/9/10 | 8/9/10 | C3 |  |  |  |  |
| 00703Dmash | GS05 | 8 | | 3 | 8a(D) | D | | 8/9/10 | 8/9/10 | C3 |  |  |  |  |
| UMB1686 | GS05 | 8 | |  |  |  | | 8/9/10 | 8/9/10 | mm |  |  |  |  |
| 6119V5 | GS05 | 9 | | 3 | 9/10(D) | D | | 8/9/10 | 8/9/10 | C3 |  |  |  |  |
| N160 | GS05 | 9* | |  |  | D | | 8/9/10 | 8/9/10 | C3 |  |  |  |  |
| 1500E | GS05 | 10 | | 3 | 9/10(D) | D | | 8/9/10 | 8/9/10 | C3 |  |  |  |  |
| GED7760B | GS06 | 11 | |  | 11 | B | |  |  |  |  |  |  |  |
| CMW7778B | GS07 | 12 | |  | 12 |  | |  |  |  |  |  |  |  |
| KA00225 | GS08 | 13 | |  | 13 |  | | Gswi/Gleo |  |  |  |  |  |  |
| UMB0768 | GS09 | 1 | |  |  |  | | 1/2 | 1/2 | C1 |  |  |  |  |
| NR010 | GS09 |  | |  |  |  | |  |  |  |  |  |  |  |
| Nine *Gardnerella genomospecies* (GS) were classified in the 1st column, based on a consensus of 4 genome wide comparison methods [28] . In the 2nd column, 13 enumerated species of *Gardnerella*, including 3 named as *G. piotti, G. swidsinski,* and *G. leopoldii*, were assigned based on average nucleotide identity (ANI), digital DNA–DNA hybridization, and MALDI-TOF mass spectroscopy protein profiling (*Gardnerella sp1* ≡ G. vaginalis) [35]. In these two columns, * indicates an isolate not used in the studies but tentatively assigned in this table, based on their placement with author-assigned species into a common branch of the NCBI genome tree (S1 Fig). The 3rd column reports results of an older study with access to fewer isolate sequences, assigned isolates to genovars based on genome wide comparison by two neighbor-joining methods of 473 aligned core open reading frames [34]. The 4th column results are based on a signature sequence within the *cpn60* gene; almost all available isolates could be assigned to the same Gsp number [69] as the genome wide assignments [35]. Scores in this column include the original group number A-D in parentheses. These *cpn60* group letters were assigned in an earlier study and are shown in the 5th column [31]. Our study constructed a phylogenetic tree based on the entire *cpn60* open reading frame (S3 Fig) and scored them in the 6th column, using Gsp nomenclature [35]. This gene does permit differentiation some Gsp species; these are indicated by pairs of species separated by /. We also scored our *cpn60* primers (S3 Table) in column 7 based on perfect complementarity to the indicated isolates, labelled as in the previous column. Clades of *Gardnerella* as determined by the clade gene-specific (CGS) qPCR numbering system [37], are reported in the 8th column. In silico scoring of the published clade gene-specific primers or probes are based on whether they were perfectly complementary to the clade gene sequences from the NCBI genome database, versus if they had one or more mismatches (mm). Blank cells in the qPCR columns indicate that the *cpn60* sequence was not available or that the gene was not found in the genomic sequence of the isolate. CGS primers for the 4 clades targeted the following genes: *fuc1* (putative α-l-fucosidase GI:311113989; C1), *hyp* (hypothetical protein GI:388060098; C2), *thi* (thioredoxin GI:388062216; C3), *cic* (Chloride transporter GI:283783343; C4) [37]. | | | | | | | | | | |  |  |  |  |
