# Supplementary material for: Prognosis of recurrent bacterial vaginosis based on longitudinal changes in abundance of Lactobacillus and specific species of Gardnerella
Source: PLoS One. 2021 Aug 23;16(8):e0256445. doi: 10.1371/journal.pone.0256445 (PMC8382169; doi:10.1371/journal.pone.0256445)
Supplement: S2 Table — Primer names reflect abbreviated versus of Gardnerella species defined by Vaneechoutte et al. [35]. Forward (F) and Reverse R) primer sequences were manually designed from alignments of cpn60 genes from isolates in the whole genome sequence at NCBI, checked for self and cross complementarity, compatible melts, and specificity with Primer Blast at NCBI against its Refseq representative genomes database and Nucleotide Blast against its Nucleotide collection (nr) and Whole genome shotgun contigs databases. 5’ position denotes the 5’-most position relative to the start of the open reading frame. Denaturation (Denat), annealing (Ann) and extension (Ext) temperatures are in °C, followed by seconds in parentheses; cycling followed an initial denaturation at 95°C for 60 s. Melt denotes melting temperature range monitored at the indicated time intervals in (seconds). Tm denotes melting temperature peaks in °C ± standard deviations encompassing sequence-verified amplicons and samples scored as positive. E denotes amplification efficiencies, derived from slopes of log molecule numbers versus Cq; correlation r values of linear regression curves were > 0.98. Molecule numbers of amplicons were determined by fluorescence assays using QuantiFluor® ONE dsDNA System with the Quantus fluorometer (Promega). Limits of detection (LOD) were estimated from molecules calculated to be present in standards or samples with the highest Cq values that had correct Tm values; for all primers, single molecules were detected in 5 to 32 samples (1–6% of total) so that LODs were estimated to be approximately 3 molecules per qPCR reaction, to give a reliable rate of detection based on the Poisson distribution. Samples reporting Cq values higher than this or incorrect Tm values were scored as negative and assigned a nominal number of molecules ten-fold lower than the limit of detection as upper limits. Primer specificities are demonstrated in the cpn60 alignment fas S1 File. (DOCX) [file pone.0256445.s006.docx]

| S2 Table. *Gardnerella* species-specific Cpn60 primers, programs, and parameters | | | | | | | | | | | |
| --- | --- | --- | --- | --- | --- | --- | --- | --- | --- | --- | --- |
| Name &  Target | **Dir** | **Sequence (5'>3’)** | **5' position** | **Amplicon bp** | **Denat** | **Ann** | **Ext** | **Cycle #** | **Melt** | **Tm** | **E** |
| Gvag.Gsp02Cpn60 | F | GTGCTGCTACGGAAGTTGAG | 1139 | 175 | 94 (20) | 58 (20) | 72 (30) | 40 | 74-92 (0.5) | 85.3 ± 0.2 | 0.87 |
|  | R | CTCTTAGTGGAGAAGAAGCC | 1314 |  |  |  |  |  |  |  |  |
| Gpio.Gsp03Cpn60 | F | CAGAAGTAGAAGCCAAGGAG | 1148 | 248 | 94 (20) | 62 (20) | 72 (30) | 45 | 74-92 (0.5) | 87.2 ± 0.3 | 0.94 |
|  | R | AGCGGTGTTTCTGGAGATGTTG | 1396 |  |  |  |  |  |  |  |  |
| Gsp08-10Cpn60 | F | GCTAAGGAGCGTAAGCATCGT | 1159 | 173 | 94 (20) | 69 (15) | 72 (30) | 45 | 74-92 (0.5) | 85.4 ± 0.2 | 1.15 |
|  | R | AACGGGCGCTGCAATCGTG | 1332 |  |  |  |  |  |  |  |  |
| Gsp07Cpn60 | F | CGCCGTGGCATCGAGAAAGCTGCT | 346 | 366 | 94 (20) | 72 (50) | | 45 | 74-92 (0.5) | 84.2 ± 0.3 | 1.07 |
| Gsp07.Gswi.GleoCpn60 | R | TATCGTCCACATCGCTGARCTTG | 712 |  |  |  |  |  |  |  |  |
|  |  |  |  | 317 |  |  |  |  |  |  |  |
| Gswi.GleoCpn60 | F | CTTCTGCAAAGGATGTTGAGACC | 395 |  | 94 (20) | 68 (20) | 72 (30) | 45 | 74-92 (0.5) | 83.9 ± 0.3 | 0.98 |
| Primer names reflect abbreviated versus of *Gardnerella* species defined by Vaneechoutte et al. [35]. Forward (F) and Reverse R) primer sequences were manually designed from alignments of Cpn60 genes from isolates in the whole genome sequence at NCBI, checked for self and cross complementarity, compatible melts, and specificity with [Primer Blast](https://www.ncbi.nlm.nih.gov/tools/primer-blast/index.cgi?LINK_LOC=BlastHome) at NCBI against its Refseq representative genomes database and [Nucleotide Blast](https://blast.ncbi.nlm.nih.gov/Blast.cgi?PROGRAM=blastn&PAGE_TYPE=BlastSearch&LINK_LOC=blasthome) against its Nucleotide collection (nr) and Whole genome shotgun contigs databases. 5’ position denotes the 5’-most position relative to the start of the open reading frame. Denaturation (Denat), annealing (Ann) and extension (Ext) temperatures are in °C followed by seconds in parentheses; cycling followed an initial denaturation at 95°C for 60 s. Melt denotes melting temperature range monitored at the indicated time intervals in (seconds). Tm denotes melting temperature peaks in °C ± standard deviations encompassing sequence-verified amplicons and samples scored as positive. E denotes amplification efficiencies, derived from slopes of log molecule numbers versus Cq; correlation r values of linear regression curves were > 0.98. Molecule numbers of amplicons were determined by fluorescence assays using QuantiFluor® ONE dsDNA System with the [Quantus](https://www.promega.com/products/microplate-readers-fluorometers-luminometers/fluorometers/quantus-fluorometer/?catNum=E6150) fluorometer (Promega). Limits of detection (LOD) were estimated from molecules calculated to be present in standards or samples with the highest Cq values that had correct Tm values; for all primers, single molecules were detected in 5 to 32 samples (1-6% of total) so that LODs were estimated to be approximately 3 molecules per qPCR reaction, to give a reliable rate of detection based on the Poisson distribution. Samples reporting Cq values higher than this or incorrect Tm values were scored as negative and assigned a nominal number of molecules ten-fold lower than the limit of detection as upper limits. Primer specificities are demonstrated in the Cpn60 alignment fas S1_file. | | | | | | | | | | | |
