# Supplementary material for: Prognosis of recurrent bacterial vaginosis based on longitudinal changes in abundance of Lactobacillus and specific species of Gardnerella
Source: PLoS One. 2021 Aug 23;16(8):e0256445. doi: 10.1371/journal.pone.0256445 (PMC8382169; doi:10.1371/journal.pone.0256445)
Supplement: S3 Table — HM numbers are references isolates obtained from BEI Resources. Clinical isolates were colony-purified from study samples and classified by sequencing their cpn60 amplicons. Gsp# [35]; GS# [28]. (DOCX) [file pone.0256445.s007.docx]

| **S3 Table. Reference and clinical isolates used to validate *cpn60* primer specificity** | | | |
| --- | --- | --- | --- |
| **Isolate** | **Isolate** | **Gsp#** | **GS#** |
| HM-1106 | JCP7276 | 1 | 1 |
| HM-1108 | JCP7672 | 1 | 1 |
| HM-1114 | JCP8108 | 2 | 1 |
| HM-1105 | JCP7275 | 2* | 1 |
| HM-1107 | JCP7659 | 3 | 2 |
| HM-1109 | JCP7719 | 3 | 2 |
| HM-1110 | JCP8017A | 3 | 2 |
| HM-1111 | JCP8017B | 3 | 2 |
| HM-1112 | JCP8066 | *G.piotii* | 2 |
| HM-1113 | JCP8070 | *G.piotii* | 2 |
| HM-1115 | JCP8151A | *G.piotii* | 2 |
| HM-1116 | JCP8151B | *G.piotii* | 2 |
| HM-1119 | JCP8522 | *G.piotii* | 2 |
| HM-1117 | JCP8481A | 7 | 4 |
| HM-1118 | JCP8481B | 7 | 4 |
| clinical isolate | C18V4-1 | 1 | 1 |
| clinical isolate | A04V4-2 | 1 | 1 |
| clinical isolate | A01V1-3 | 3 | 2 |
| clinical isolate | A05V5-1 | *Gsp08,Gsp09, & Gsp10* | 5 |
| clinical isolate | A41V1-2 | 7 | 4 |
| clinical isolate | C18V4-5 | *G.swidsinskii, G. leopoldii* | 3 |
| HM numbers are references isolates obtained from [BEI Resources](https://www.beiresources.org/Home.aspx). Clinical isolates were colony-purified from study samples and classified by sequencing their *cpn60* amplicons. | | | |
